# Food‐Herb Dual‐Function in Astragali Radix‐Poria‐Rheum: Network Pharmacology and Database Mining for Diabetic Kidney Disease Mechanisms Exploration

**DOI:** 10.1002/fsn3.71251

**Published:** 2025-11-27

**Authors:** Meizi Wang, Weizhong Li, Qiong Cheng, Jiayu Sun, Cheng Cheng, Nana Wang, Ping Zhou, Min Zha, Yufeng Yang

**Affiliations:** ^1^ Jiangsu Province Hospital of Chinese Medicine Affiliated Hospital of Nanjing University of Chinese Medicine Nanjing China; ^2^ School of Health Preservation and Rehabilitation Nanjing University of Chinese Medicine Nanjing China; ^3^ School of Nursing Nanjing University of Chinese Medicine Nanjing China; ^4^ Liaoning University of Traditional Chinese Medicine Shenyang Liaoning China

**Keywords:** functional foods, glycolipid metabolism, insulin resistance, medicinal and edible homology, metabolic diseases

## Abstract

The growing demand for functional foods derived from medicinal‐edible homologous resources offers a promising strategy to address the chronic metabolic dysregulation underlying diabetic kidney disease (DKD), particularly in patients requiring long‐term pharmacological interventions. DKD, a metabolic disorder‐driven complication of diabetes, is characterized by chronic hyperglycemia, insulin resistance (IR), and systemic metabolic dysregulation, necessitating novel dietary approaches to complement conventional therapies. To systematically explore the therapeutic potential of medicinal‐edible homologous herbs in DKD management, this study retrieved clinical literature from the China National Knowledge Infrastructure (CNKI) database, focusing on traditional Chinese medicines (CHMs) with dual dietary and therapeutic functions. By integrating data mining techniques (frequency analysis, attribute‐flavor analysis, herb‐pair co‐occurrence networks, and cluster analysis) with expert clinical consensus, we prioritized the identification of core medicinal‐edible homologous combinations that align with DKD's metabolic pathology. This multi‐dimensional approach revealed Huangqi (*Astragali Radix*), Fuling (*Poria*), and Dahuang (*Rhei Radix et Rhizoma*) (Ast‐Por‐Rhe) as the most predictive herb combination, highlighting their synergistic role as functional food‐derived agents in mitigating DKD progression. Network pharmacology revealed that Ast‐Por‐Rhe's bioactive components (e.g., isorhamnetin, 7‐O‐methyleriodictyol) act as dual‐purpose nutrients and metabolic regulators, synergistically targeting 38 DKD‐associated genes. Functional enrichment analysis demonstrated Ast‐Por‐Rhe's capacity to restore metabolic homeostasis by alleviating IR through dephosphorylation modulation, while rectifying PI3K‐Akt signaling. In a streptozotocin (STZ)‐induced DKD rat model, Ast‐Por‐Rhe supplementation, as a functional food intervention, significantly mitigated renal metabolic dysfunction, evidenced by enhanced IRS1/PI3K/Akt activity, reduced lipid peroxidation, and improved renal fibrosis. These findings position Ast‐Por‐Rhe as a paradigm of medicinal‐edible homologous functional foods that counteract DKD progression through multi‐component synergy, specifically targeting IR‐driven metabolic perturbations. This study provides a translational roadmap for developing evidence‐based dietary strategies using bioactive food components, advancing the application of medicinal‐edible resources in metabolic disorder management.

## Graphical abstract

This study identified the core food‐herb formula “Astragali Radix‐Poria‐Rheum (Ast‐Por‐Rhe)” for Diabetic Kidney Disease (DKD) through data mining of clinical prescriptions. Integrated network pharmacology and in vivo validation revealed that this formula ameliorates renal injury and insulin resistance in DKD rats by activating the IRS1/PI3K/AKT signaling pathway. Our findings provide a scientific basis for this formula as a potential complementary therapy for DKD.

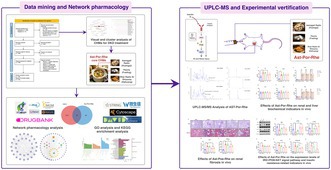

**Abbreviations**
AKT: serine–threonine kinase
ALT: serum alanine transaminase
Ast‐Por‐Rhe: Huangqi (*Astragali Radix*), Fuling (*Poria*), and Dahuang (*Rhei Radix et Rhizoma*)
BG: blood glucose
BUN: blood urea nitrogen
CHMs: Chinese medicines
CNKI: China National Knowledge Infrastructure
DKD: diabetic kidney disease
ESRD: end‐stage renal disease
GCP: glomerular cell population
GO: gene ontology
GS: glomerular sclerosis
IR: insulin resistance
IRS1: insulin receptor substrate 1
KEGG: Kyoto Encyclopedia of genes and genomes
p‐AKT: phosphorylated serine–threonine kinase
PAS: periodic acid‐Schiff
PI3K: phosphoinositide 3‐kinase
PPI: protein–protein interaction
p‐PI3K: phosphorylated phosphoinositide 3‐kinase
Scr: serum creatinine
STZ: streptozotocin
TCM: traditional Chinese medicine
UAlb: urinary albumin

## 1 Introduction

Diabetic kidney disease (DKD), a metabolic complication of type 2 diabetes, has emerged as the leading cause of end‐stage renal disease (ESRD) worldwide, driven by progressive renal fibrosis and irreversible functional decline (Kidney Disease: Improving Global Outcomes (KDIGO) CKD Work Group 2024; Perkovic et al. 2024). Currently, the management of DKD has been transformed by the introduction of novel agents such as Sodium‐Glucose Cotransporter‐2 (SGLT2) inhibitors and Glucagon‐Like Peptide‐1 Receptor Agonists (GLP‐1 RAs). These drugs have demonstrated robust evidence in improving renal outcomes and slowing disease progression (Bae 2025). While current therapeutic approaches partially slow disease progression, their efficacy in ameliorating metabolic dysregulation—particularly insulin resistance (IR) remains suboptimal. Recent studies highlight IR not merely as a peripheral metabolic disorder but as a pivotal pathological mechanism identifiable in early‐stage DKD, wherein impaired insulin signaling exacerbates glomerulosclerosis and tubular injury through oxidative stress, chronic inflammation, and aberrant lipid metabolism (Adeva‐Andany et al. 2023; Penno et al. 2021). Given the persistent limitations of current clinical therapies in effectively addressing IR in DKD, there is an urgent imperative to develop functional foods grounded in the medicinal‐food homology theory of traditional Chinese medicine (TCM), which integrate bioactive therapeutic properties with inherent nutritional safety, thereby providing a sustainable approach to ameliorate DKD progression and reduce hepatorenal toxicity burdens in patients requiring lifelong treatment.

Medicinal‐edible homologous resources, recognized as natural functional foods in traditional dietary systems, offer a unique advantage in targeting IR‐driven metabolic dysregulation (Lyu et al. 2022). Common dietary ingredients with medicinal‐food homologous properties, such as *Dioscoreae Rhizoma* (Shanyao), *Ziziphi Fructus* (Dazao), and *Mori Folium* (Sangye), exemplify functional foods enriched with bioactive compounds, including dioscin and flavonoids (e.g., quercetin, rutin). These phytochemicals exhibit multi‐target biological activities, notably antioxidant, anti‐inflammatory, and antimicrobial effects, which collectively contribute to their therapeutic potential in preventing chronic metabolic disorders. Studies have demonstrated that dioscin, a bioactive steroidal saponin derived from Dioscorea species, alleviates oxidative stress and inflammatory responses by modulating the RAGE/NOX4 signaling pathway, thereby suppressing ROS overproduction and NF‐κB‐mediated cytokine release (Guan et al. 2022). Concurrently, *Ziziphus jujuba* (jujube) polyphenols exhibit anti‐inflammatory properties through the NO/cGMP pathway activation, which downregulates oxidative stress‐associated biomarkers and enhances endothelial nitric oxide synthase (eNOS) activity (Shaukat et al. 2022). Furthermore, mulberry leaf polyphenols demonstrate antihyperglycemic and lipid‐lowering effects through AMPK‐dependent mechanisms, underscoring their role in holistic dietary strategies for metabolic syndrome management (Li et al. 2020). This convergence of nutritional and pharmacological properties highlights the translational value of medicinal‐edible homologous resources as sustainable, evidence‐based interventions in public health nutrition (Green et al. 2020). Notably, medicinal‐edible homologous therapy demonstrates unique advantages in clinical practice: characterized by mild pharmacological properties, it alleviates the hepatorenal burden associated with prolonged medication use, rendering it particularly suitable for DKD patients requiring lifelong management.

Emerging computational approaches have accelerated the discovery of bioactive‐driven functional foods from medicinal‐edible homologous systems, particularly for managing metabolic disorders such as DKD (Wu et al. 2021; Yoo et al. 2012). Recent advances in data mining have unveiled the pivotal role of pharmaco‐dietary herbs in TCM, particularly through frequency analysis and prescription pattern recognition (Sattari and Mohammadi 2023; Tang et al. 2021). This study aims to identify core herbal combinations from clinical prescriptions of TCM for DKD using data mining techniques, with a specific focus on prioritizing medicinal‐edible homologous herbs and decoding their therapeutic potential through multi‐target metabolic regulation, bioactive synergy, and safety profiles suitable for long‐term dietary interventions.

**FIGURE 1:**
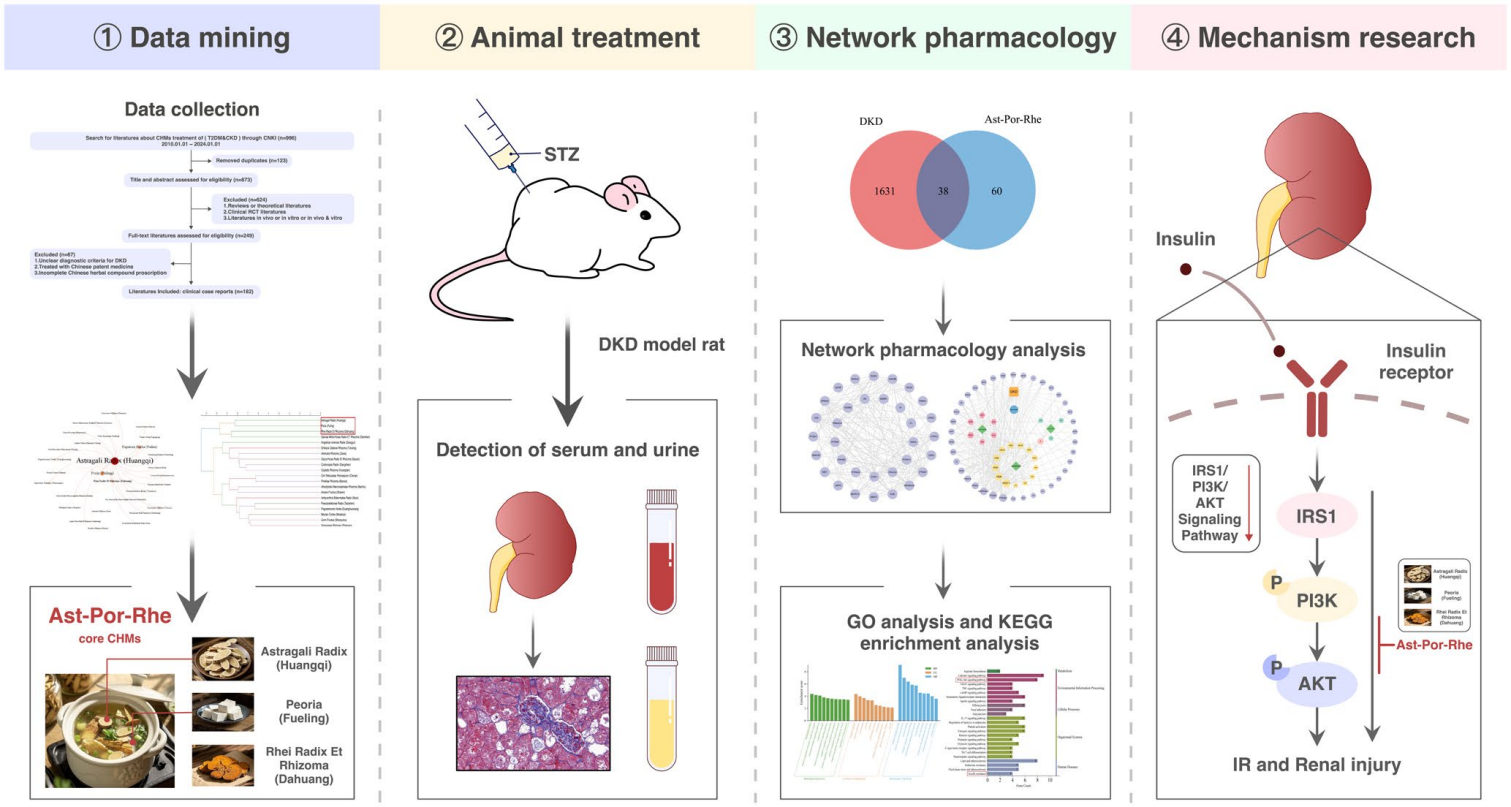
Flowchart of this study.

Studies demonstrate that over 70% of core herbs (e.g., *Astragalus membranaceus*, *Poria*) used in DKD management are listed in China's pharmaco‐dietary catalogue, functioning primarily via spleen‐kidney tonification and dampness‐heat resolution. Building on this foundation, our study innovatively integrates network pharmacology and experimental validation to decode the “food‐to‐medicine” transition mechanism (Guo et al. 2025; Liu et al. 2024; Shen et al. 2023). Our study innovatively integrates clinical data mining with network analysis to: (1) Identify core pharmaco‐dietary CHMs through prescription pattern analysis; (2) Construct ingredient‐target‐pathway networks to elucidate their scientific rationality in DKD intervention; (3) Validate the renal protective effects using core medicinal food‐derived formulation in DKD rat models. This tripartite approach not only deciphers the “preventive treatment of disease progression” principle embodied in food‐medicine combinations but also facilitates the development of dietary supplement strategies targeting early‐stage DKD. The experimental design workflow is systematically presented in Figure 1.

## 2 Materials and Methods

### 2.1 Data Mining

#### 2.1.1 Literature Search

We searched China National Knowledge Infrastructure (CNKI) regardless of language from the year of establishment of the database. The literature search was from January 1, 2010 to January 1, 2024. The main search strategy included “the key words” (Diabetic kidney disease/Type 2 Diabetes + chronic kidney disease/Type 2 Diabetes + chronic renal failure/Type 2 Diabetes + chronic renal insufficiency) AND (academic thought) OR (experience), “the theme” (Chinese medicine) OR (traditional Chinese medicine). “Time” was used as sorting mode and cooperated with manual search at the same time. The relevant original data can be found in Appendix S1

#### 2.1.2 Criteria for Considering Studies

##### 2.1.2.1 Inclusion Criteria

(1) The patients of any age and of both sexes with clinically diagnosed DKD; (2) All selected cases were case reports, case series and doctors' personal clinical experiences; (3) All selected cases included Chinese herbal compound prescriptions and clinical data. Here, the literature could be included when the above 3 conditions were both met.

##### 2.1.2.2 Exclusion Criteria

(1) The literature about basic research, theoretical studies, reviews and clinical randomized controlled trials (RCT); (2) The duplicate literature; (3) The incomplete data.

#### 2.1.3 Data Collection

**FIGURE 2:**
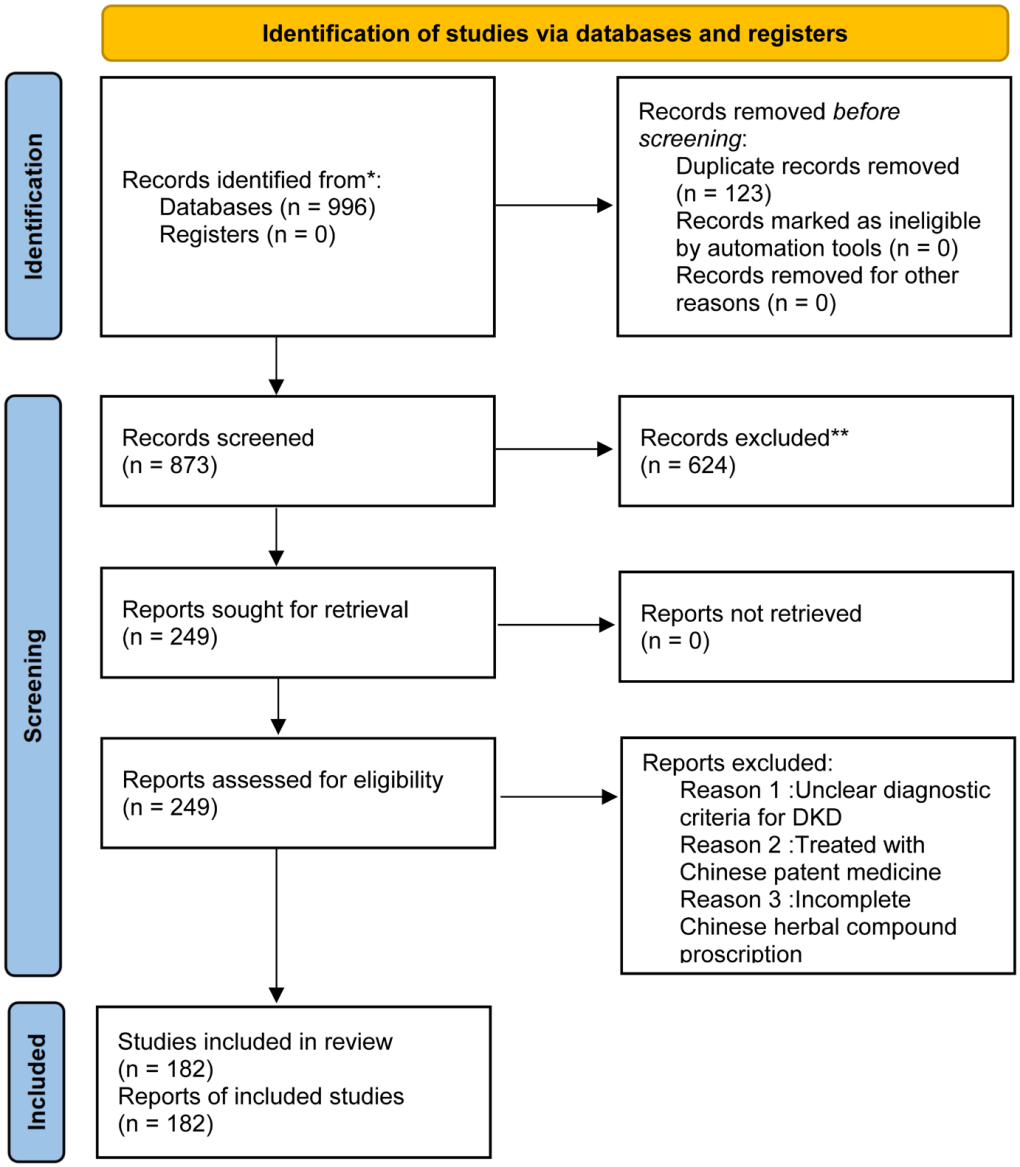
PRISMA flow diagram illustrating the literature identification and screening process for data mining. Adapted from: Page et al. (2021). This work is licensed under CC BY 4.0. To view a copy of this license, visit https://creativecommons.org/licenses/by/4.0/.

Two senior researchers (Dr. Wang and Dr. Li) double‐checked the results of literature searches and entered the 182 cases into an Excel table. To ensure the accuracy and completeness of the data, inconsistencies were resolved by 1 senior researcher (Dr. Zha). The literature screening process followed the PRISMA reporting guidelines (Page et al. 2021), as illustrated in the flow diagram Figure 2. A total of 996 records were initially identified, from which 182 studies were ultimately included for data mining after a systematic screening process. For the specific standardized process, please refer to Appendix S2.

#### 2.1.4 Data Analysis

Ancient and Modern Medical Record Cloud Platform System V2.1 (V2.1) was used to analyze the clinical medication rules of CHMs. This system was designed especially for data mining analysis. In this study, the function modules of medication and multi‐dimensional analysis in the special software (V2.1) were used to analyze the frequencies and attributes of CHMs. Meanwhile, association rules analysis and cluster analysis were used to discover rules of CHMs compatibility. In addition, we standardized the name of CHMs in the process of data entry according to the *Pharmacopeia of the People's Republic of China (2015 Edition)*. For instance, “Kuncao” (*Leonuri Herba*) was unified as “Xianyimucao” (*Leonuri Herba*).

### 2.2 Network Pharmacology

#### 2.2.1 Screening Potential Active Compounds and Targets of Ast‐Por‐Rhe

Utilize the Traditional Chinese Medicine Systems Pharmacology Database and Analysis Platform (TCMSP, https://old.tcmsp‐e.com/tcmsp.php) to search for the chemical constituents of Ast‐Por‐Rhe (Huangqi, Fuling, and Dahuang) related Chinese herbal medicines. Perform preliminary screening of active components based on two ADME (Absorption, Distribution, Metabolism, and Excretion) parameters: oral bioavailability (OB) ≥ 30% and drug likeness (DL) ≥ 0.18 to identify active compounds and their corresponding protein targets. After the screening process, standardize the information of the protein targets of the compounds through the UniProt protein database (https://www.uniprot.org/).

#### 2.2.2 Identification of Potential Targets of Ast‐Por‐Rhe in Treating DKD

First, the disease targets information related to DKD was searched and gathered using the keyword “diabetic kidney disease” through three databases: OMIM (https://www.omim.org/), Genecards (https://www.genecards.org/), DisGeNet (http://www.disgenet.org/), and DrugBank (https://go.drugbank.com/). Next, the unique DKD‐related targets from the above step were combined with the compound targets retrieved from the TCMSP database. Both datasets were imported into the bioinformatics online platform (https://www.bioinformatics.com.cn/) to identify their intersection, thereby pinpointing the potential targets of Ast‐Por‐Rhe in treating DKD.

#### 2.2.3 Construction of PPI Network and “Ast‐Por‐Rhe‐Active Ingredient‐DKD‐Target” Network

Submit the intersecting targets to the STRING 11.0 database (https://cn.string‐db.org/), setting the organism to “Homo sapiens” to obtain protein–protein interaction (PPI) network information. Utilize Cytoscape 3.9.0 software to further analyze and visualize the PPI network and the drug active component–targeted disease target network.

#### 2.2.4 GO Enrichment and KEGG Pathway Analysis

Perform GO function and KEGG pathway enrichment analysis on the Ast‐Por‐Rhe‐DKD intersecting targets using the DAVID database (https://david.ncifcrf.gov/tools.jsp). The GO function analysis includes three parts: biological process (BP), cellular component (CC), and molecular function (MF).

### 2.3 Animal Experiments

#### 2.3.1 Materials

Ast‐Por‐Rhe was prepared by The Affiliated Hospital of Nanjing University Medical School (Nanjing, China). The Whole Cell Lysis Assay was sourced from KeyGEN BioTECH. Antibodies against insulin receptor substrate 1 (IRS1), phosphorylated phosphoinositide 3‐kinase (p‐PI3K), phosphorylated serine–threonine kinase (p‐AKT), phosphoinositide 3‐kinase (PI3K), serine–threonine kinase (AKT), β‐actin were acquired from Cell Signaling Technology. Malondialdehyde (MDA) Assay Kit (M496) from Dojindo Molecular Technologies Inc.

#### 2.3.2 Animal Grouping and Model Establishment

Male Sprague–Dawley (SD) rats (200–220 g) were purchased from the SPF (Beijing, China) Biotechnology Co. Ltd. (License No: SCXK [Beijing] 2019‐0010). All rats were housed (six rats/cage) at 22°C ± 3°C and 50% ± 10% humidity using a 12 h light/dark cycle, fed a specific pathogen‐free grade standard rat chow (catalogue number 1010008) from Xietong Pharmaceutical Bio‐engineering Co. Ltd. (Nanjing, China), and provided tap water ad libitum in the Experimental Animal Centre of Nanjing University of Chinese Medicine. The animal ethics committee of Nanjing University of Chinese Medicine approved the surgical procedures and protocols (SBK2024044934). SD rats were randomly assigned to one of five groups: (1) Sham, (2) Vehicle, (3) low‐dose (L), (4) medium‐dose (M), and (5) high‐dose (H), using a random number table. Personnel responsible for outcome assessments were blinded to the group allocations throughout the experiment. Researchers induced DKD in rats through a combination of high‐fat diet feeding, unilateral nephrectomy, and STZ administration. After 4 weeks on a high‐fat diet, rats underwent a left unilateral nephrectomy under isoflurane anesthesia. One week post‐surgery, diabetes was induced by two intraperitoneal injections of STZ (35 mg/kg). Diabetic rats (random blood glucose > 16.7 mmol/L) were maintained with insulin injections if necessary to prevent severe hyperglycemia. The DKD model was considered successfully established 5 weeks later upon confirmation of significantly increased urinary albumin and renal function indicators. Rats that did not meet all the established criteria were excluded from the study, yielding a final count of five rats per group for all subsequent experiments (Wang et al. 2022). Based on the human daily dosage (Astragalus 30 g, Poria 15 g, and Rhei Radix et Rhizoma 10 g), the equivalent rat dose was calculated using the human‐to‐rat body surface area conversion factor. The calculated base doses were as follows: Astragalus 540 mg·kg^−1^, Poria 270 mg·kg^−1^, and Rhei Radix et Rhizoma 180 mg·kg^−1^, resulting in a total base dose (for the Ast‐Por‐Rhe) of 990 mg·kg^−1^. Accordingly, the administered doses for each treatment group were set as follows: the L group received 495 mg·kg^−1^, the M group received 990 mg·kg^−1^, and the H group received 1980 mg·kg^−1^. The herbal mixture was prepared to appropriate concentrations in normal saline and administered by oral gavage once daily for 4 consecutive weeks. The Sham and Vehicle groups received an equal volume of normal saline via oral gavage. After 4 weeks of treatment with different intervention measures, all rats were anesthetized and euthanized by cardiac puncture. Blood, urine and kidneys were collected for testing various analyses.

#### 2.3.3 Chemical Component Profile Analysis of Ast‐Por‐Rhe

The chemical composition of Ast‐Por‐Rhe was evaluated using the ExionLC AD system coupled with a QTRAP mass spectrometer, which was fitted with an electrospray ionization (ESI) source, provided by SCIEX (USA). A gradient elution was programmed as follows: the proportion of acetonitrile (B) increased from 15% to 95% between 1 and 25 min, was maintained at 95% for 1 min, and then returned to the initial condition for column re‐equilibration.

#### 2.3.4 Biochemical Parameters

Blood glucose (BG) levels were assessed prior to modeling and biweekly thereafter. After 4 weeks of drug intervention, the rats were anesthetized, and blood samples (5 mL) were extracted from the abdominal aorta. A range of biochemical parameters, including serum creatinine (Scr), blood urea nitrogen (BUN), serum alanine transaminase (ALT), and serum aspartate transaminase (AST) levels, were measured. Prior to sacrifice, urinary samples were collected from each of the five groups for the assessment of 24 h urinary albumin (UAlb) levels.

#### 2.3.5 Insulin Resistance‐Related Indicators

Prior to sacrifice, the rats in all five groups were fasted for 8 h, after which fasting blood glucose and fast insulin (FINS) levels were measured. The insulin resistance index for each group was calculated using the formula HOMA‐IR = FBG × FINS/22.5, as reported in the literature (Matthews et al. 1985).

#### 2.3.6 Renal MDA Content

The lipid peroxidation level in renal tissue was evaluated by measuring the content of MDA using a commercial assay kit according to the manufacturer's instructions. Briefly, approximately 30 mg of renal tissue was homogenized in 500 μL of ice‐cold Antioxidant PBS. The homogenate was then centrifuged at 12,000 rpm for 5 min at 4°C to collect the supernatant. Subsequently, a 200 μL aliquot of the supernatant was mixed with 200 μL of Lysis Buffer and incubated at room temperature for 5 min. Following this, 300 μL of the Working solution was added to the mixture, which was then vortexed and heated in a 95°C water bath for 15 min. After cooling on ice for 5 min, the sample was centrifuged again at 12,000 rpm for 10 min at 4°C. Finally, 200 μL of the resulting supernatant was transferred to a 96‐well plate, and its absorbance was measured at a wavelength of 532 nm using a microplate reader. The MDA concentration was calculated based on a standard curve.

#### 2.3.7 Histological Analysis

Following euthanasia, kidney tissue samples were fixed in 4% paraformaldehyde and embedded in paraffin. Sections of 3 μm thickness were cut perpendicularly to the long axis of the kidney for morphometric analyses. For histological analysis, the sections were stained with periodic acid‐Schiff (PAS) staining and Masson's trichrome staining. The kidney sections were examined using light microscopy (LM). Five sections (PAS staining) from each group were randomly selected to calculate glomerular cell population (GCP) per glomerulus by IPP. Analogously, five sections (Masson staining) from each group were randomly selected to calculate the area of collagen per glomerulus by IPP. These results were confirmed by a professional pathologist.

#### 2.3.8 Western Blot

After the animals were sacrificed, kidney tissue samples were collected, and stored at −80°C until testing. Weigh the tissue, cut it into small pieces and put it into 1.5 mL EP tubes, and prepare a protein lysis reagent containing protease and phosphatase inhibitors. Add inhibitor‐containing protein lysis reagent to the 1.5 mL EP tubes. Use a homogenizer to crush the tissue 6 times until the tissue is completely lysed. After complete crushing, lyse on ice for 30 min. Then put it into a pre‐cooled centrifuge at 12,000 rpm, 4°C, for 25 min. Discard the precipitate, take part of the supernatant, measure the protein concentration and calculate the dilution concentration. After adding 5× loading buffer to the remaining supernatant, place it in a water bath at 95°C for 10 min to completely denature the protein. The proteins were separated using sodium dodecyl sulfate polyacrylamide gel electrophoresis and transferred onto polyvinylidene fluoride membranes. After blocking with 5% skim milk at room temperature, each membrane was incubated with IRS1, p‐PI3K, p‐AKT, PI3K, AKT, and β‐actin primary antibodies overnight at 4°C, followed by incubation with Goat Anti Rabbit IgG‐HRP for 1 h at room temperature. The membranes were washed three times with TBST after each incubation. The protein bands were visualized using the Tanon Chemiluminescent Imaging System and band intensities were analyzed with ImageJ gel analysis software.

#### 2.3.9 Statistical Analysis

All data were expressed as mean ± standard deviation (SD). The data obtained were analyzed using GraphPad Prism 8 software. Differences among groups were assessed using one‐way analysis of variance (ANOVA), with a significance level set at *p* < 0.05.

## 3 Results

### 3.1 Frequency Analysis

The 182 Chinese herbal compound prescriptions for DKD treatment were entered through V2.3, respectively, and a total of 177 CHMs for DKD treatment were extracted. Further, the frequency of CHMs and prescriptions was counted. The frequency of CHMs was ranked from high to low; therein, the top 5 CHMs were *Astragali Radix* (Huangqi), *Poria* (Fuling), *Rhei Radix Et Rhizoma* (Dahuang), *Salviae Miltiorrhizae Radix ET Rhizoma* (Danshen) and *Atractylodis Macrocephalae Rhizoma* (Baizhu). The top 20 CHMs are shown in Table 1.

**TABLE 1:** Frequency statistics of the top 20 CHMs for DKD treatment.

No.	CHMs Latin name (Chinese name)	Frequency	Rate (%)
1	*Astragali Radix* (Huangqi)	133	73.08
2	*Poria* (Fuling)	111	60.99
3	*Rhei Radix Et Rhizoma* (Dahuang)	95	52.20
4	*Salviae Miltiorrhizae Radix ET Rhizoma* (Danshen)	87	47.80
5	*Atractylodis Macrocephalae Rhizoma* (Baizhu)	74	40.66
6	*Citri Reticulatae Pericarpium* (Chenpi)	71	39.01
7	*Angelicae sinensis Radix* (Danggui)	67	36.81
8	*Pinelliae Rhizoma* (Banxia)	64	35.16
9	*Amomi Fructus* (Sharen)	63	34.62
10	*Corni Fructus* (Shanyurou)	60	32.97
11	*Smilacis Glabrae Rhizoma* (Tufuling)	57	31.32
12	*Pseudostellariae Radix* (Taizishen)	55	30.22
13	*Dioscoreae Rhizoma* (Shanyao)	54	29.67
14	*Codonopsis Radix* (Dangshen)	46	25.27
15	*Pogostemonis Herba* (Guanghuoxiang)	45	24.73
16	*Achyranthris Bidenntatae Radix* (Niuxi)	41	22.53
17	*Moutan Cortex* (Mudanpi)	40	21.98
18	*Coptidis Rhizoma* (Huanglian)	40	21.98
19	*Alismatis Rhizoma* (Zexie)	39	21.43
20	*Glycyrrhizae Radix Et Rhizoma* (Gancao)	39	21.43

### 3.2 Attribute Analysis

**FIGURE 3:**
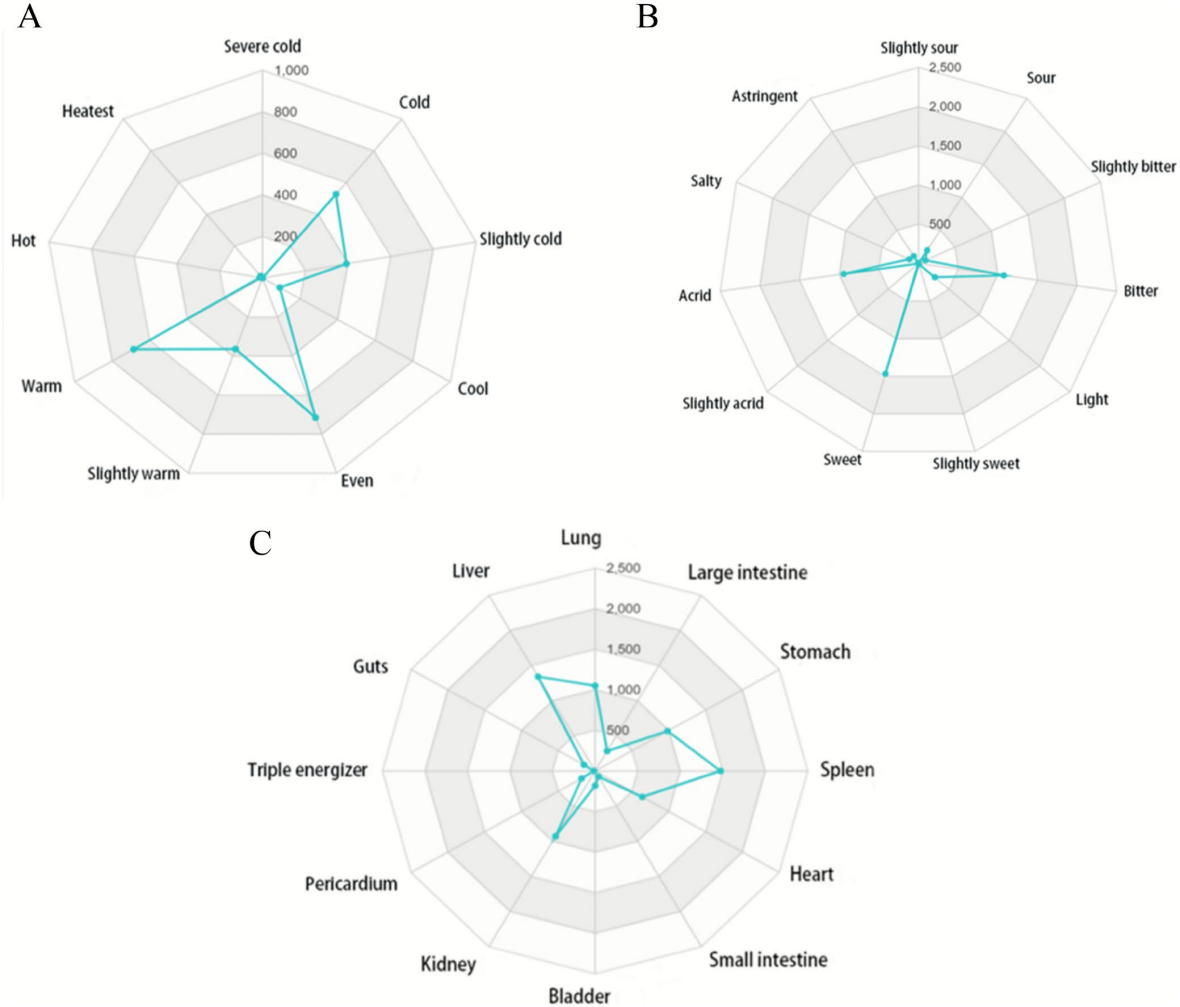
Analysis of CHMs' natures, flavors, and meridian tropism in the treatment of DKD. (A) Radar map of four natures of CHMs for DKD treatment. (B) Radar map of five flavors of CHMs for DKD treatment. (C) Radar map of meridian tropism of CHMs for DKD treatment.

Four natures of CHMs for DKD treatment mainly included even (715), warm (686) and cold (527). The frequency statistics were shown in Table 2, and the related radar map was shown in Figure 3A. Five flavors of CHMs for DKD treatment mainly included sweet (1467), bitter (1075) and acrid (943). The frequency statistics were shown in Table 3, and the related radar map was shown in Figure 3B. Meridian tropism of CHMs for DKD treatment mainly included spleen (1475), liver (1343), lung (1050), stomach (984) and kidney (930). The frequency statistics were shown in Table 4, and the related radar map was shown in Figure 3C.

**TABLE 2:** Frequency statistics of four natures of CHMs for DKD treatment.

Four natures	Frequency
Even	715
Warm	686
Cold	527
Slightly cold	393
Slightly warm	364
Cool	92
Heatest	13
Heat	12

**TABLE 3:** Frequency statistics of five flavors of CHMs for DKD treatment.

Five flavors	Frequency	Five flavors	Frequency
Sweet	1467	Salty	128
Bitter	1075	Astringent	111
Acrid	943	Slight bitter	93
Light	269	Slight sweet	10
Sour	198	Slight acrid	2

**TABLE 4:** Frequency statistics of meridian tropism of CHMs for DKD treatment.

Meridian tropism	Frequency	Meridian tropism	Frequency
Spleen	1475	Large intestine	283
Liver	1343	Pericardium	183
Lung	1050	Bladder	182
Stomach	984	Guts	151
Kidney	930	Small intestine	80
Heart	640	Triple energizer	14

### 3.3 Co‐Occurrence CHMs Pairs Analysis

Association rules reflecting the interdependence and association between 2 things are an important technology of data mining analysis. The compatibility of CHMs is the main form of clinical medication; we thereby used the Apriori algorithm to analyze association rules of CHMs pairs, which is of great significance in clinics. In association rules, Lift reflects the correlation of 2 factors. When Lift is more than 1, the bigger the better. Otherwise, there is no correlation. According to the analysis of association rules, the co‐occurrence CHMs pairs for DKD treatment were screened with Lift > 1, and the critical values of Confidence and Support were 0.74 and 0.1. Here, as a result, a total of 45 CHMs pairs were obtained in Table 5. In addition, the top 4 CHMs pairs with the co‐occurrence frequency were all compatible with *Astragali Radix* (Huangqi). Therefore, the 4 common co‐occurrence CHMs pairs for DKD treatment were *Astragali Radix* (Huangqi) and *Poria* (Fuling), *Astragali Radix* (Huangqi) and *Rhei Radix Et Rhizoma* (Dahuang), *Astragali Radix* (Huangqi) and *Salviae Miltiorrhizae Radix ET Rhizoma* (Danshen), *Astragali Radix* (Huangqi) and *Atractylodis Macrocephalae Rhizoma* (Baizhu).

**TABLE 5:** Co‐occurrence CHMs pairs for DKD treatment (Confidence ≥ 0.74, Support ≥ 0.1, Lift > 1).

No.	Herbs	Herbs	Co‐occurrence frequency	Confidence	Support	Lift
1	*Poria* (Fuling)	*Astragali Radix* (Huangqi)	88	0.79	0.48	1.08
2	*Rhei Radix Et Rhizoma* (Dahuang)	*Astragali Radix* (Huangqi)	71	0.75	0.39	1.03
3	*Salviae Miltiorrhizae Radix ET Rhizoma* (Danshen)	*Astragali Radix* (Huangqi)	65	0.75	0.36	1.03
4	*Atractylodis Macrocephalae Rhizoma* (Baizhu)	*Astragali Radix* (Huangqi)	60	0.81	0.33	1.11
5	*Citri Reticulatae Pericarpium* (Chenpi)	*Poria* (Fuling)	56	0.79	0.31	1.3
6	*Amomi Fructus* (Sharen)	*Astragali Radix* (Huangqi)	54	0.86	0.3	1.18
7	*Corni Fructus* (Shanyurou)	*Astragali Radix* (Huangqi)	52	0.87	0.29	1.19
8	*Pinelliae Rhizoma* (Banxia)	*Poria* (Fuling)	50	0.78	0.27	1.28
9	*Pseudostellariae Radix* (Taizishen)	*Astragali Radix* (Huangqi)	48	0.87	0.26	1.19
10	*Pinelliae Rhizoma* (Banxia)	*Citri Reticulatae Pericarpium* (Chenpi)	48	0.75	0.26	1.92
11	*Dioscoreae Rhizoma* (Shanyao)	*Astragali Radix* (Huangqi)	48	0.89	0.26	1.22
12	*Corni Fructus* (Shanyurou)	*Poria* (Fuling)	45	0.75	0.25	1.23
13	*Dioscoreae Rhizoma* (Shanyao)	*Poria* (Fuling)	44	0.81	0.24	1.33
14	*Smilacis Glabrae Rhizoma (Tufuling)*	*Astragali Radix* (Huangqi)	43	0.75	0.24	1.03
15	*Pogostemonis Herba* (Guanghuoxiang)	*Astragali Radix* (Huangqi)	37	0.82	0.2	1.12
16	*Pogostemonis Herba* (Guanghuoxiang)	*Rhei Radix Et Rhizoma* (Dahuang)	36	0.8	0.2	1.53
17	*Cuscutae Semen* (Tusizi)	*Astragali Radix* (Huangqi)	35	0.92	0.19	1.26
18	*Achyranthris Bidenntatae Radix* (Niuxi)	*Astragali Radix* (Huangqi)	31	0.76	0.17	1.04
19	*Cuscutae Semen* (Tusizi)	*Pseudostellariae Radix* (Taizishen)	31	0.82	0.17	2.71
20	*Cuscutae Semen* (Tusizi)	*Rhei Radix Et Rhizoma* (Dahuang)	31	0.82	0.17	1.57
21	*Alismatis Rhizoma* (Zexie)	*Poria* (Fuling)	31	0.79	0.17	1.3
22	*Moutan Cortex* (Mudanpi)	*Salviae Miltiorrhizae Radix ET Rhizoma* (Danshen)	30	0.75	0.16	1.57
23	*Chuanxiong Rhizoma* (Chuanxiong)	*Astragali Radix* (Huangqi)	30	0.81	0.16	1.11
24	*Plantaginis Semen* (Cheqianzi)	*Poria* (Fuling)	29	0.76	0.16	1.25
25	*Ostreae Concha* (Muli)	*Astragali Radix* (Huangqi)	26	0.93	0.14	1.27
26	*Fry Atractylodis Macrocephalae Rhizoma* (Chaobaizhu)	*Astragali Radix* (Huangqi)	26	0.84	0.14	1.15
27	*Eupatorii Herba* (Peilan)	*Pogostemonis Herba* (Guanghuoxiang)	25	0.96	0.14	3.88
28	*Eupatorii Herba* (Peilan)	*Astragali Radix* (Huangqi)	24	0.92	0.13	1.26
29	*Eupatorii Herba* (Peilan)	*Rhei Radix Et Rhizoma* (Dahuang)	24	0.92	0.13	1.76
30	*Liquor Rhei Radix Et Rhizoma* (Jiudahuang)	*Astragali Radix* (Huangqi)	24	0.75	0.13	1.03
31	*Rehmanniae Radix Praeparata* (Shudihuang)	*Astragali Radix* (Huangqi)	23	0.79	0.13	1.08
32	*Imperatae Rhizoma* (Baimaogen)	*Astragali Radix* (Huangqi)	23	0.85	0.13	1.16
33	*Eupatorii Herba* (Peilan)	*Pseudostellariae Radix* (Taizishen)	23	0.88	0.13	2.91
34	*Cortex Eucommiae* (Duzhong)	*Astragali Radix* (Huangqi)	22	0.88	0.12	1.2
35	*Ostreae Concha* (Muli)	*Poria* (Fuling)	21	0.75	0.12	1.23
36	*Taraxaci Herba* (Pugongying)	*Astragali Radix* (Huangqi)	21	0.81	0.12	1.11
37	*Imperatae Rhizoma* (Baimaogen)	*Rhei Radix Et Rhizoma* (Dahuang)	21	0.78	0.12	1.49
38	*Eupatorii Herba* (Peilan)	*Cuscutae Semen* (Tusizi)	21	0.81	0.12	3.88
39	*Eupatorii Herba* (Peilan)	*Atractylodis Macrocephalae Rhizoma* (Baizhu)	21	0.81	0.12	1.99
40	*Imperatae Rhizoma* (Baimaogen)	*Poria* (Fuling)	20	0.74	0.11	1.21
41	*Eupatorii Herba* (Peilan)	*Corni Fructus* (Shanyurou)	20	0.77	0.11	2.34
42	*Eupatorii Herba* (Peilan)	*Amomi Fructus* (Sharen)	20	0.77	0.11	2.22
43	*Leonuri Herba* (Xianyimucao)	*Astragali Radix* (Huangqi)	20	0.83	0.11	1.14
44	*Leonuri Herba* (Xianyimucao)	*Poria* (Fuling)	20	0.83	0.11	1.36
45	*Paeoniae Radix Rubra* (Chishao)	*Astragali Radix* (Huangqi)	18	0.78	0.1	1.07

### 3.4 Visual and Cluster Analysis of CHMs for DKD Treatment

**FIGURE 4:**
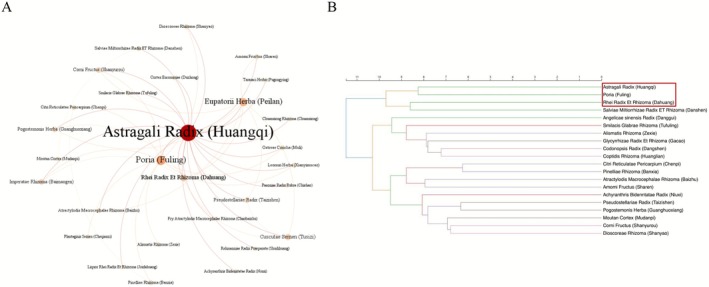
Exploring association rules and clustering patterns. (A) Complex network analysis of association rules (the confidence degree: 0.74). (B) Dendrogram of cluster analysis.

We used the Visual Analysis Software Gephi to make a visual analysis of the co‐occurrence CHMs pairs under association rules. According to the confidence degree of 0.74, we got the associated networks showing the relationship among CHMs for DKD treatment dynamically. In Figure 4A, each circle represented a node, the location and size of the node reflected the importance and component proportion of the items respectively, and the line between the nodes represented the association between the items. The different colors were used to better distinguish the strength of the relationship between the nodes. As shown in the network graph, we found that *Astragali Radix* (Huangqi) was the central node in color blue. Moreover, according to the node area, *Astragali Radix* (Huangqi) had the largest area, followed by *Poria* (Fuling), *Macrocephalae Rhizoma* (Baizhu) and *Rhei Radix Et Rhizoma* (Dahuang). In brief, in response to high‐frequency CHMs, the 3 predicted core CHMs for CRF treatment based on visualized analysis were *Astragali Radix* (Huangqi), *Poria* (Fuling) and *Rhei Radix Et Rhizoma* (Dahuang).

Further, we applied cluster analysis to categorize the top 20 CHMs by frequency using the Euclidean distance method. In Figure 4B, each category was represented by a different color. We found that CHMs for DKD treatment could be divided into 4 groups based on the special Euclidean distance (> 9). The first group included *Astragali Radix* (Huangqi) and *Poria* (Fuling); the second group included *Rhei Radix Et Rhizoma* (Dahuang) and *Salviae Miltiorrhizae Radix ET Rhizoma* (Danshen). The third group included *Angelicae sinensis Radix* (Danggui), *Smilacis Glabrae Rhizoma* (Tufuling), *Alismatis Rhizoma* (Zexie), *Glycyrrhizae Radix Et Rhizoma* (Gancao), *Codonopsis Radix* (Dangshen) and *Coptidis Rhizoma* (Huanglian). Here, the fourth group was divided into two groups again with the special Euclidean distance (= 8), as the limit. Therein, one group was *Citri Reticulatae Pericarpium* (Chenpi), *Pinelliae Rhizoma* (Banxia), *Atractylodis Macrocephalae Rhizoma* (Baizhu) and *Amomi Fructus* (Sharen); another group was *Achyranthris Bidentatae Radix* (Niuxi), *Pseudostellariae Radix* (Taizishen), *Pogostemonis Herba* (Guanghuoxiang), *Moutan Cortex* (Mudanpi), *Corni Fructus* (Shanyurou) and *Dioscoreae Rhizoma* (Shanyao). In addition, we also found that the first two groups of CHMs summarized by cluster analysis were highly consistent with the top 4 CHMs with the highest frequency of ranking in frequency analysis. In short, in response to high‐frequency CHMs, the 3 predicted core CHMs for DKD treatment based on cluster analysis were *Astragali Radix* (Huangqi), *Poria* (Fuling) and *Rhei Radix Et Rhizoma* (Dahuang).

### 3.5 Analysis of Targets of Ast‐Por‐Rhe in the Treatment of DKD

**FIGURE 5:**
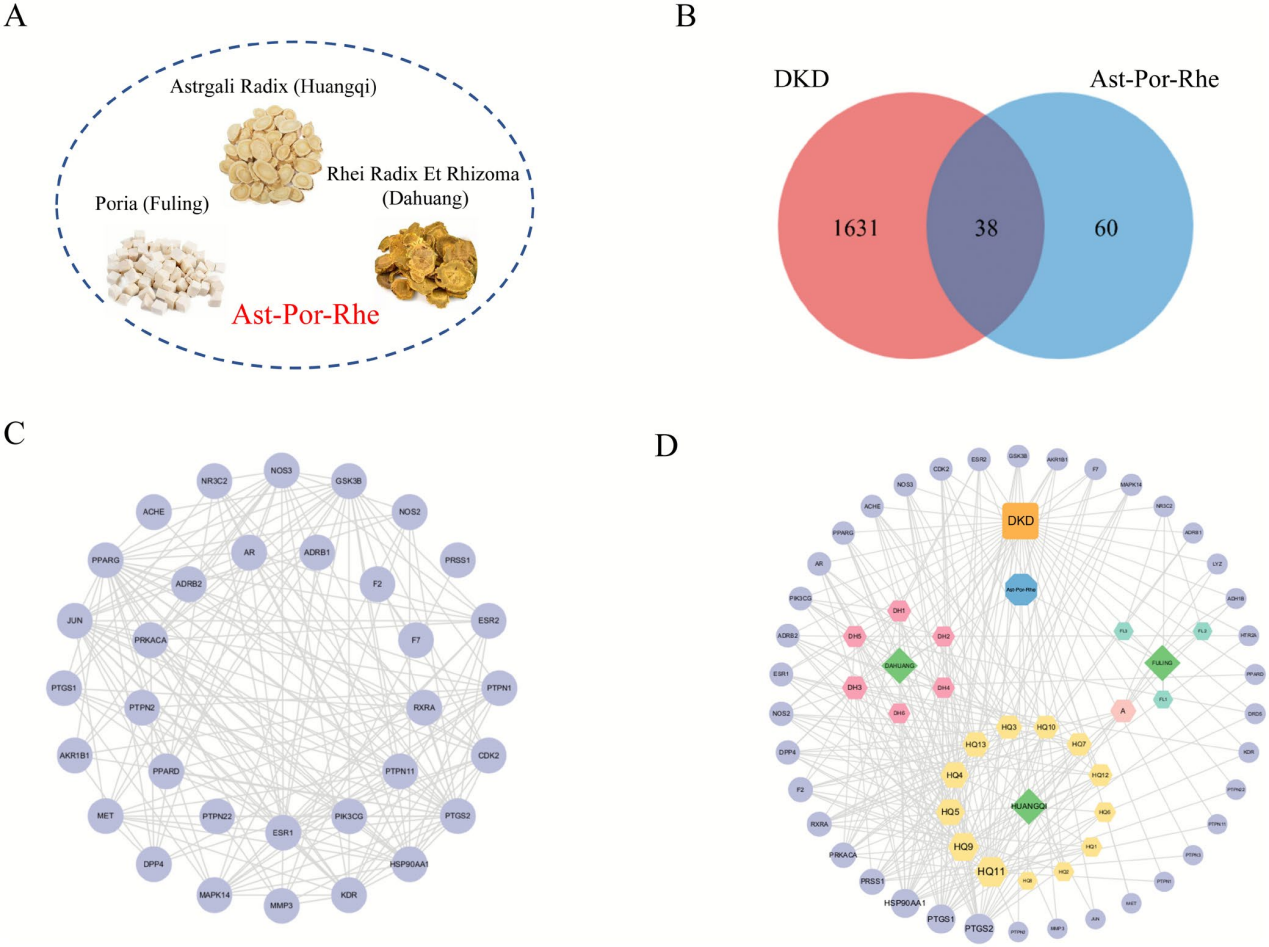
Targets of Ast‐Por‐Rhe in the treatment of DKD. (A) Medicinal components of Ast‐Por‐Rhe. (B) Intersection targets of Ast‐Por‐Rhe active ingredients and DKD. (C) PPI network of Ast‐Por‐Rhe in the treatment of DKD. (D) Network of Ast‐Por‐Rhe active components and their targeted DKD targets. Rounded rectangle‐disease, octagon‐drug, rhombus‐medicinal material, hexagon‐active ingredient, circle‐target.

In the TCMSP database, active components in Ast‐Por‐Rhe (Figure 5A) were screened with the criteria of OB ≥ 30% and DL ≥ 0.18, followed by target prediction. After standardizing and integrating the target names and removing duplicates, 98 active component targets for Ast‐Por‐Rhe were obtained. Specifically, 20 active components were identified for Astragali Radix, 15 for Poria, and 16 for Rheum. After deduplication of the above components, a total of 50 unique active components were obtained. Subsequently, the target names corresponding to these 50 unique active components were standardized and integrated, and duplicates were removed. Finally, 30 of these unique active components were found to map to valid targets, resulting in 98 active component targets for Ast‐Por‐Rhe. A total of 1669 DKD targets were retrieved. A total of 1669 DKD targets were retrieved through databases such as GeneCards, OMIM, Disgenet, and DrugBank. The intersection of the Ast‐Por‐Rhe active component targets and DKD‐related targets was obtained using the bioinformatics drawing platform, resulting in 38 common targets for “Ast‐Por‐Rhe‐DKD,” as shown in Figure 5B. Subsequently, the 38 intersection targets were submitted to the String 11.0 platform, and the PPI network diagram was visualized using Cytoscape 3.9.1 software, as shown in Figure 5C. Then, the relationship network of Ast‐Por‐Rhe active components and their targeted DKD disease‐related targets was visualized and analyzed using Cytoscape 3.9.0 software, yielding 66 nodes and 270 relationships, as shown in Figure 5D. Network Analyzer was used to analyze the network topological parameters of active components and targets, where the Degree value reflects the involvement of a node in the network, with a higher value indicating greater importance. The network topological parameters of the active components are shown in Table 6, and the results show that Isorhamnetin and 7‐O‐Methylisomucronulatol have higher Degree values.

**TABLE 6:** Topological analysis results of main active components of Ast‐Por‐Rhe (top 10 in degree).

	Herbs	MOL ID	Molecule name	Degree
HQ11	Huangqi	MOL000354	Isorhamnetin	26
HQ9	Huangqi	MOL000378	7‐O‐Methylisomucronulatol	22
HQ5	Huangqi	MOL000392	Formononetin	18
HQ4	Huangqi	MOL000417	Calycosin	17
HQ13	Huangqi	MOL000098	Quercetin	15
HQ3	Huangqi	MOL000422	Kaempferol	13
DH3	Dahuang	MOL002235	Eupatin	12
HQ10	Huangqi	MOL000371	3,9,10‐Trimethoxypterocarpan	12
A	Huangqi, Fuling	MOL000296	Hederagenin	12
HQ7	Huangqi	MOL000380	Methylnissolin	11

### 3.6 GO and KEGG Pathway Enrichment Analysis

**FIGURE 6:**
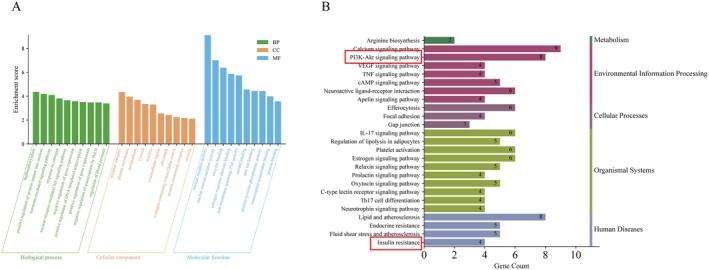
GO analysis and KEGG enrichment analysis. (A) GO analysis. (B) KEGG enrichment analysis.

The obtained 38 intersection targets were imported into the DAVID data platform for GO functional and KEGG pathway enrichment analysis. On the basis of a *p*‐value < 0.01, 51 BP (Biological Process), 11 CC (Cellular Component), and 29 MF (Molecular Function) terms were selected. The results indicate that the active components of Ast‐Por‐Rhe primarily act on cellular structures such as the plasma membrane, nucleus, and chromatin; molecular functions include the regulation of nuclear receptor activity, protein tyrosine phosphatase activity, etc.; biological processes include dephosphorylation, gene expression, DNA transcription, etc. The enrichment results were visualized, as shown in Figure 6A. Based on a *p*‐value < 0.01, 29 KEGG terms were selected, indicating that the target points are mainly enriched in pathways such as the calcium ion signaling pathway and the PI3K‐Akt signaling pathway. The enrichment results were visualized, as shown in Figure 6B. These pathways play an important role in the signal regulation pathways of DKD renal fibrosis.

### 3.7 UPLC‐MS/MS Analysis of Ast‐Por‐Rhe

**FIGURE 7:**
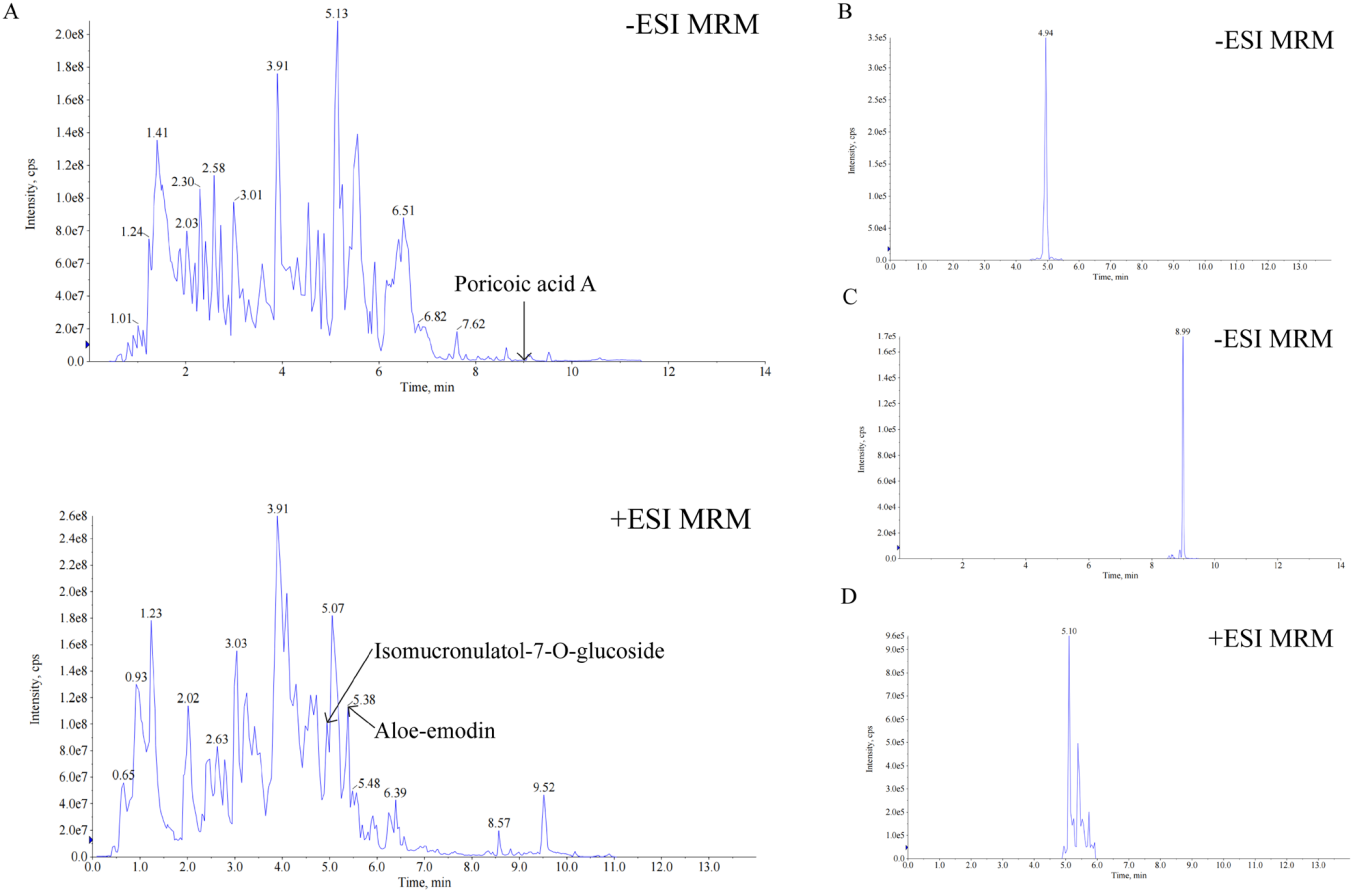
UPLC‐MS/MS Analysis of Ast‐Por‐Rhe. (A) TIC of Ast‐Por‐Rhe in Positive and Negative Ion Modes. (B) Representative XIC of characteristic bioactive compounds in Astragali Radix. (C) Representative XIC of characteristic bioactive compounds in Poria. (D) Representative XIC of characteristic bioactive compounds in Rheum.

The Total Ion Chromatograms (TIC) chromatograms obtained in both negative and positive MRM modes exhibited multiple peaks across the analytical run, indicating the complex composition of the sample (Figure 7A). The results demonstrate that the sample derived from the combination of Astragalus, Poria, and Rhubarb is rich in various chemical constituents in both positive and negative ion modes. The analysis of extracted ion chromatograms (XIC) confirmed the presence of three target compounds by matching their experimental retention times with the expected values (Figure 7B–D).

### 3.8 Ast‐Por‐Rhe Ameliorated Renal Function in vivo

**FIGURE 8:**
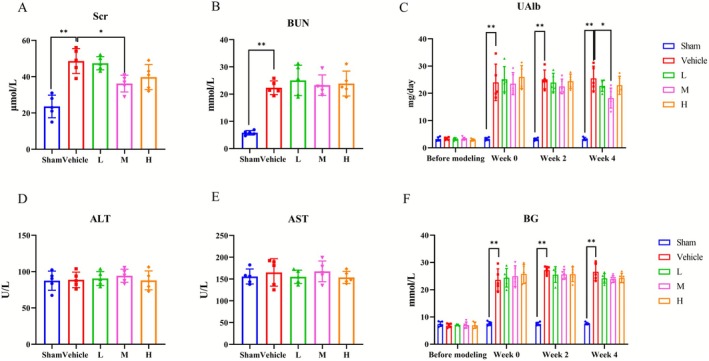
Effects of Ast‐Por‐Rhe on renal and liver biochemical indicators in vivo. (A, B) The levels of Scr and BUN. (C) The dynamic changes in UAlb after drug intervention during 4 weeks. (D, E) The levels of ALT and AST. (F) The dynamic changes in BG after drug intervention during 4 weeks. The data are expressed as the mean ± SD. * *p* < 0.05; ** *p* < 0.01.

As shown in Figure 8A,B, Ast‐Por‐Rhe treatment exhibited varying degrees of reduction in Scr levels across the groups, with a significant decrease observed only in the M group in comparison with the Vehicle group. However, BUN levels did not show a substantial decrease. In addition, as shown in Figure 8C, after 4 weeks of Ast‐Por‐Rhe treatment, the elevated UAlb levels in DKD model rats were significantly reduced in the M group compared to the Vehicle group. As shown in Figure 8D,E, there was no significant difference in ALT and AST levels among all groups. Notably, in Figure 8F, we found that Ast‐Por‐Rhe did not influence hyperglycemia in the DKD model rats.

### 3.9 Ast‐Por‐Rhe Alleviated Renal Fibrosis in vivo

**FIGURE 9:**
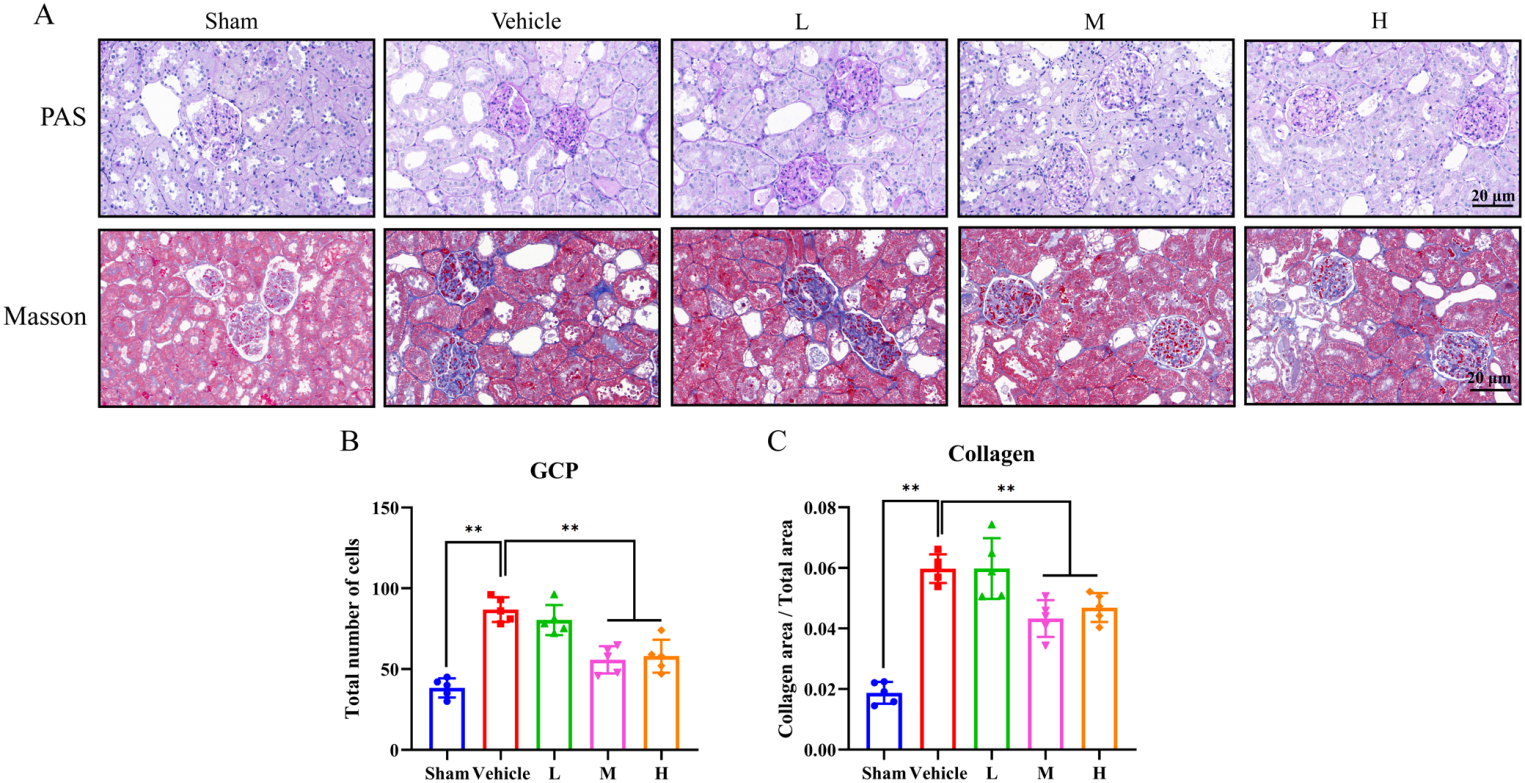
Effects of Ast‐Por‐Rhe on renal fibrosis in vivo. (A) The staining of PAS and Masson stains in the diabetic kidneys (×400). Scale bar = 20 μm. (B) The number of GCP. (C) The rate of collagen area/glomerular area. ** *p* < 0.01.

Renal fibrosis in DKD is predominantly characterized by glomerular sclerosis (GS), with key indices including glomerular cell proliferation (GCP) and collagen area within the glomerulus. Thus, we investigated the impact of Ast‐Por‐Rhe on GS in DKD model rats. As shown in Figure 9, we found that, significant pathological alterations were observed in the Vehicle group, such as mild glomerular hypertrophy, reduced capillary loop area, increased glomerular cell proliferation, augmented glomerular matrix, and collagen deposition. Following treatment with the M group and H group, the pathological severity of GS, as indicated by GCP and the collagen area to glomerular area ratio, was notably alleviated compared to the Vehicle group.

### 3.10 Ast‐Por‐Rhe Regulated the IRS1/PI3K/AKT Signal Pathway and Attenuated Insulin Resistance in vivo

**FIGURE 10:**
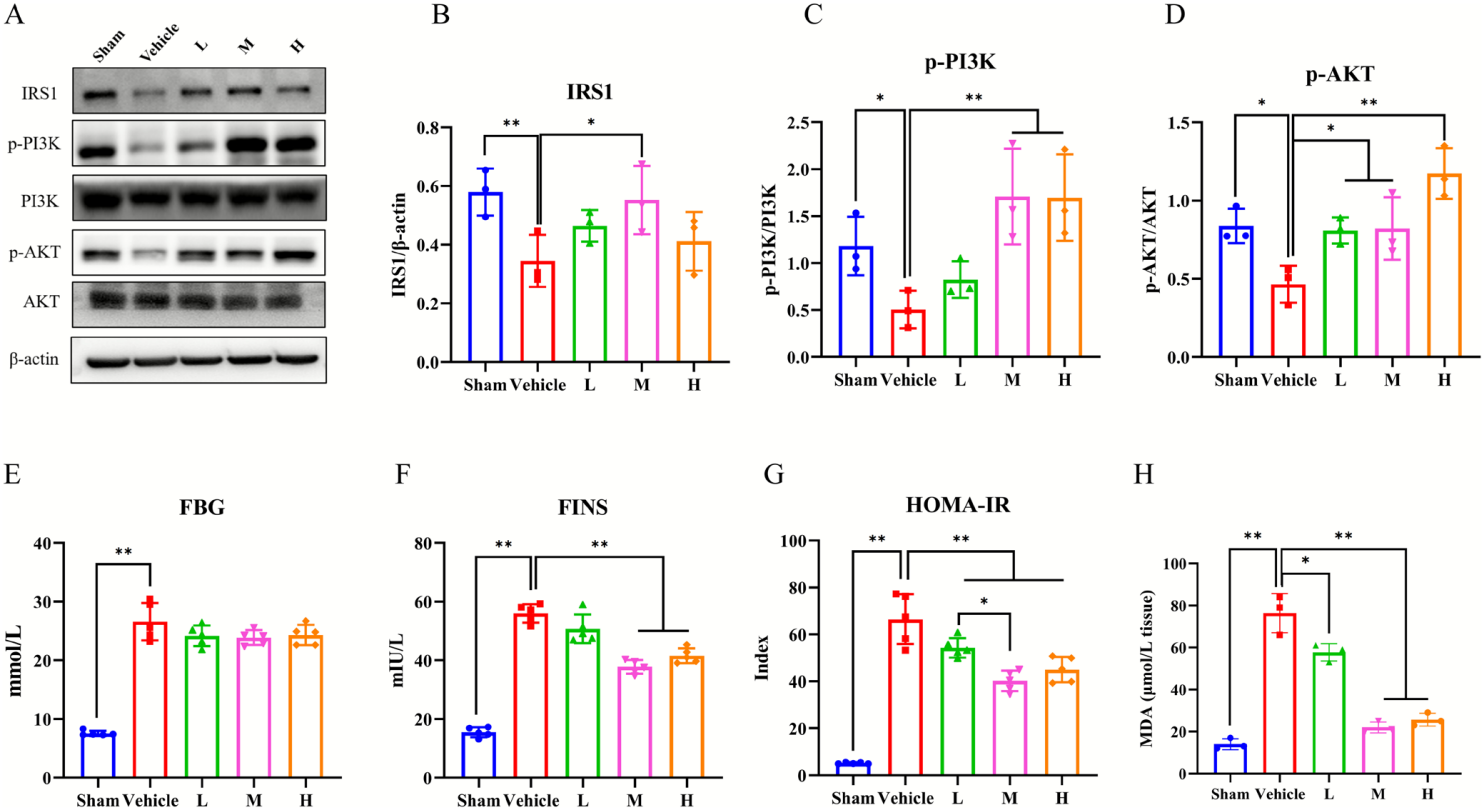
Effects of Ast‐Por‐Rhe on the expression levels of IRS1/PI3K/AKT signal pathway and insulin resistance‐related indicators and oxidative stress (MDA) in vivo. (A) The WB analysis of IRS1, PI3K and AKT in the diabetic kidneys. (B) The rate of IRS1 to β‐Actin. (C) The rate of p‐PI3K to PI3K. (D) The rate of p‐AKT to AKT. (E–G) The levels of FBG, FINS and HOMA‐IR. (E and H) The levels of MDA. The data are expressed as the mean ± SD. * *p* < 0.05; ** *p* < 0.01.

To elucidate the mechanisms of Ast‐Por‐Rhe improving DKD, we quantified the relative expression levels of key proteins in the IRS1/PI3K/AKT signaling pathway, including IRS1, p‐PI3K, PI3K, p‐AKT and AKT, using western blot analysis. As shown in Figure 10A–D, Ast‐Por‐Rhe treatment led to increased expression levels of IRS1, p‐PI3K/PI3K, and p‐AKT/AKT to varying extents, with significant upregulation observed exclusively in the M group compared to the Vehicle group. Furthermore, as shown in Figure 10E–G, after treatment with Ast‐Por‐Rhe, FBG, FINS, and HOMA‐IR levels were decreased to varying degrees. FINS levels were significantly reduced in both the M and H groups in comparison with the Vehicle group, and HOMA‐IR levels were significantly decreased in all three Ast‐Por‐Rhe dose groups compared to the Vehicle group. The extent of lipid peroxidation in renal tissue, as assessed by the measurement of MDA levels, is presented in Figure 10H. As expected, the renal MDA content in the Sham‐operated group remained at a low baseline level. In contrast, the Vehicle model group resulted in a significant elevation of MDA levels, indicating a pronounced state of oxidative stress. Treatment with the Ast‐Por‐Rhe formula effectively ameliorated this oxidative damage in a dose‐dependent manner. Compared to the Vehicle group, the M and H groups exhibited a significant and progressive reduction in MDA content. The L group also showed a decreasing trend, though the difference did not reach statistical significance. Notably, the MDA levels in the M group were reduced to a level comparable to that of the Sham group, suggesting a potent anti‐lipid peroxidative effect of the core formula.

## 4 Discussion

Focusing on the unique advantages of medicinal–food homology in DKD management, data mining has emerged as a sophisticated methodology for extracting implicit patterns from massive datasets and has demonstrated unique value in inheriting TCM clinical expertise (Liu et al. 2013; Teng et al. 2016; Wu et al. 2021). This study systematically deciphered the medication patterns for DKD treatment through multidimensional analytical approaches, including frequency analysis, association rule mining, and complex network modeling. It is worth noting that cluster analysis in this study further identified that the 3 predicted core CHMs for DKD treatment were *Astragali Radix* (Huangqi), *Poria* (Fuling) and *Rhei Radix Et Rhizoma* (Dahuang). *Astragali Radix* (Huangqi) and *Poria* (Fuling) exhibit the characteristic of “medicinal and edible homology”, embodying the distinctive TCM philosophy integrating preventive treatment of disease and disease progression control (Hu et al. 2022; Wu and Hu 2020). These dual‐purpose substances, officially listed in both the Chinese Pharmacopeia and the National Health Commission's Catalogue of Medicinal‐Food Homology Materials, provide novel insights for developing stepped therapeutic strategies in DKD management due to their established safety profiles and feasibility for long‐term application (Leong et al. 2020; Xu et al. 2021).

*Astragali Radix* (Huangqi) a canonical medicinal food in TCM, has been revered as the “supreme herb for replenishing Qi (vital energy)” for over 2000 years (Dai et al. 2022). Its dried root, characterized by a sweet flavor and warm nature with meridian tropism targeting the spleen and lungs, is widely utilized for both therapeutic and dietary purposes (Tian et al. 2020). Clinically, it is prescribed to address Qi deficiency syndromes, including fatigue, spontaneous sweating, night sweats, and edema. In traditional culinary practices, Astragalus root is frequently incorporated into decoctions or soups combined with nutrient‐rich ingredients such as aged hen, *Ziziphi Fructus* (Dazao), and *Lycii Fructus* (Gouqi). This synergy not only amplifies its tonic effects but also imparts a naturally sweet flavor profile to the dish, aligning with the principles of food therapy in TCM.

*Poria* (Fuling), a saprophytic fungus that grows symbiotically on pine roots, is characterized by its dried sclerotium, which exhibits a mild flavor and neutral nature in TCM, with meridian tropism targeting the heart, spleen, and kidneys (Duan et al. 2023). When rehydrated, Fuling develops a subtle earthy sweetness and a slightly chewy texture, making it an ideal culinary additive that enhances mouthfeel without dominating other ingredients. Its blandness allows it to absorb and harmonize flavors in both savory and sweet dishes, contributing to its widespread use in daily diets (Kim et al. 2023; Zhao et al. 2023). While strengthening spleen function and promoting dampness drainage in pharmacological applications, it concurrently regulates metabolic homeostasis as a dietary component.

Pharmacological investigations have validated that bioactive constituents in *Astragali Radix*, particularly polysaccharides, exhibit multifaceted therapeutic effects, including immunomodulatory activity, diuretic effects, antioxidant capacity, and anti‐inflammatory properties (Huang et al. 2012; Nalbantsoy et al. 2012; Qin et al. 2012). Moreover, modern pharmacological studies have validated that *Poria* polysaccharides not only ameliorate renal oxidative stress (Jiang et al. 2022), but its dietary fiber constituents also reduce uremic toxin accumulation by modulating gut microbiota composition (Tan et al. 2022). This multi‐target characteristic aligns with the holistic regulatory mechanism inherent to medicinal‐edible homologous substances. *Rhei Radix Et Rhizoma* (Dahuang) and its components emodin and rhein also have assured effects in DKD treatment, and their mechanisms include many ways such as ameliorating azotemia, preventing nephritic compensatory hypertrophy and high metabolism situations, and so on (Lian et al. 2014; Yang et al. 2018). The medicinal‐edible homologous properties of these substances confer distinct therapeutic advantages across different stages of DKD management.

To explore the mechanism of Ast‐Por‐Rhe in treating DKD, this study explored the theoretical basis of Ast‐Por‐Rhe's effects on DKD using network pharmacology. Through the TCMSP database of drug active ingredients and targets, as well as the DrugBank, GeneCards, OMIM, and DisGeNet databases of disease targets, we identified a total of 38 potential targets for the treatment of DKD with Ast‐Por‐Rhe. The construction of the “Ast‐Por‐Rhe‐Active Ingredients‐DKD‐Targets” network revealed that Ast‐Por‐Rhe exhibits a multi‐component and multi‐target characteristic in the treatment of DKD. Among these, Isorhamnetin and 7‐O‐Methylisomucronulatol are considered particularly significant active ingredients.

Further GO and KEGG enrichment analyses indicated that these target sites primarily involve dephosphorylation, the regulation of protein tyrosine phosphatase activity, and insulin resistance, among other biological processes and functions. Notably, they are significantly enriched in the PI3K‐Akt signaling pathway. Research has pointed out that insulin resistance is a critical feature of type 2 diabetes, closely associated with elevated blood glucose levels. The long‐term damage to the kidneys caused by hyperglycemia is a significant trigger for the onset and progression of diabetic kidney disease (Kanwar et al. 2011).

This section delineates the pivotal role of IRS1 and the PI3K/Akt signaling pathway in the transmission of insulin signals, as elucidated by extant research. Specifically, upon the binding of insulin to its receptor, the receptor's intrinsic tyrosine kinase is activated, catalyzing the phosphorylation of tyrosine residues. This event subsequently triggers the phosphorylation of the IRS (Insulin Receptor Substrate) family, with IRS1 predominantly engaging the PI3K subunit to activate PI3K, thereby facilitating the phosphorylation of Akt (Boucher et al. 2014; Copps and White 2012). Consequently, we hypothesize that Ast‐Por‐Rhe primarily exerts its beneficial effects on DKD by modulating the IRS1/PI3K/Akt signaling pathway.

To further clarify the mechanism of Ast‐Por‐Rhe in treating DKD, this study experimentally confirmed Ast‐Por‐Rhe's therapeutic action and potential mechanisms in the rat model. We employed a modified rat model of DKD induced through a comprehensive approach involving high‐fat diet administration, unilateral nephrectomy, and intraperitoneal injection of STZ. Post‐induction, the model rats exhibit not only a significant elevation in renal injury markers (Scr, BUN, UAlb) but also the development of fibrosis characterized by glomerulosclerosis. Following intervention with Ast‐Por‐Rhe administered via gavage at low, medium, and high doses, the renal injury markers and glomerulosclerosis in the model rats are ameliorated, with the exception of BUN and BG. Notably, the medium‐dose Ast‐Por‐Rhe group demonstrates superior efficacy compared to the low and high‐dose groups.

The kidney, functioning as a “target organ for insulin receptor,” exhibits insulin resistance when its sensitivity and responsiveness to endogenous or exogenous insulin diminish. Under such conditions, the activation of IRS1 may be compromised, subsequently affecting the IRS1/PI3K/AKT signaling pathway. Research has indicated a close correlation between insulin resistance and kidney injury in DKD (Horita et al. 2016). In this study, the expression levels of IRS1, p‐PI3K, and p‐AKT proteins in the renal tissues of DKD model rats were significantly downregulated, while the indices of IR were markedly upregulated. Following intervention with Ast‐Por‐Rhe, not only did the activity of the IRS1/PI3K/AKT pathway in the renal tissues of the model rats recover, but the IR indicators (FBG, FINS, HOMA‐IR) also improved. Similar to its impact on renal injury, the M group exhibited superior effects in ameliorating IR and the IRS1/PI3K/AKT signaling pathway compared to the low and high dose groups.

This study demonstrates that Ast‐Por‐Rhe, a representative medicinal‐food homologous formulation, exerts its renoprotective effects in DKD through IRS1/PI3K/AKT pathway activation. Notably, Ast‐Por‐Rhe's dual therapeutic–dietary properties enable effective modulation of insulin resistance and renal fibrosis progression while maintaining superior safety profiles—a critical advantage for long‐term DKD management compared to conventional mono‐target therapies.

## 5 Conclusion

In summary, this study identified a “core herbal formula” (Ast‐Por‐Rhe) through clinical data mining that incorporates the principles of “medicinal‐food homology”– a unique feature of CHMs where therapeutic ingredients also serve as dietary components. Our network pharmacology analysis revealed that Ast‐Por‐Rhe's mechanism against DKD particularly leverages its dual medicinal‐nutritional properties to modulate the PI3K/Akt signaling pathway. Experimental validation demonstrated that this food‐derived therapeutic formula alleviates renal fibrosis by synergistically regulating insulin resistance and the IRS1/PI3K/Akt pathway activity through its nutrient‐pharmacological components. Notably, the medicinal‐food homologous characteristics of Ast‐Por‐Rhe components ensure both therapeutic efficacy and nutritional safety, making it particularly suitable for long‐term management of chronic conditions like DKD. These findings not only elucidate the scientific basis of CHMs in DKD treatment but also highlight the distinctive advantages of medicinal‐food homologous therapies in achieving renal protection through dietary intervention strategies.

## 6 Limitations

It should be noted that this study has several limitations. First, the findings were derived from the frequency analysis of CHMs documented in published literature, and the clinical efficacy of individual herbs remains to be systematically validated. Second, the therapeutic mechanisms underlying the three predicted core CHMs for DKD treatment require further elucidation through molecular biology and multi‐omics approaches. Third, the current data mining methodology necessitates refinement to enhance analytical precision.

Future investigations will prioritize the exploration of targeted interactions between medicinal‐food homologous components and their protein targets, aiming to systematically elucidate the molecular mechanisms of the herbal formula (Ast‐Por‐Rhe). Although this study has not fully delineated the molecular basis of Ast‐Por‐Rhe's renoprotective effects in DKD, our findings establish a novel research paradigm for medicinal‐food homologous therapies. This integrative approach, combining traditional herbal knowledge with modern biotechnology, provides a theoretical foundation for developing dietary interventions that synergistically integrate nutritional support with targeted therapeutic effects in chronic disease management.

## Author Contributions

M.W.: conceptualization, data curation, formal analysis, methodology, project administration, validation, writing – original draft. W.L.: data curation, formal analysis, methodology, project administration, writing – original draft. Q.C.: data curation, project administration, writing – original draft. J.S.: data curation, formal analysis, project administration, writing – original draft. C.C.: data curation, methodology, funding acquisition, writing – original draft. N.W.: conceptualization, resources, writing – review editing. P.Z.: project administration, resources, writing – review editing. M.Z.: conceptualization, funding acquisition, investigation, resources, writing – review editing. Y.Y.: conceptualization, methodology, resources, writing – review editing.

## Conflicts of Interest

The authors declare no conflicts of interest.

## Supporting information


**Appendix S1:** fsn371251‐sup‐0001‐AppendixS1.xlsx.

**Appendix S2:** fsn371251‐sup‐0002‐AppendixS2.docx.

## Data Availability

The original contributions presented in this study are included in the article/Supporting Information. Further inquiries can be directed to the corresponding author(s).
